# The modulation of steady-state responses by transcranial alternating current stimulation: a scoping review

**DOI:** 10.3389/fnsys.2025.1661128

**Published:** 2025-10-20

**Authors:** Aurimas Mockevičius, Jovana Bjekić, Inga Griškova-Bulanova

**Affiliations:** ^1^Institute of Bioscience, Life Sciences Center, Vilnius University, Vilnius, Lithuania; ^2^Faculty of Medicine, Translational Health Research Institute, Vilnius University, Vilnius, Lithuania; ^3^Centre for Neuroscience and Neuromodulation, Institute for Medical Research, University of Belgrade, Belgrade, Serbia

**Keywords:** steady state response, auditory steady state response, steady state visual evoked potential, transcranial alternating current stimulation, non-invasive brain stimulation

## Abstract

Transcranial alternating current stimulation (tACS) is a non-invasive technique that modulates brain oscillatory activity in a frequency-specific manner, offering potential for improving sensory and cognitive functions. Steady-state responses (SSRs), which are periodic neural responses to rhythmic sensory stimulation, provide a robust and objective means to assess tACS effects. The present work systematically reviews the existing literature on tACS modulation of SSR. 16 studies that used either auditory (ASSR) or visual (SSVEP) SSR were included in the review. Findings indicate that tACS can enhance or suppress SSRs depending on stimulation parameters. Although ASSR studies reported mixed findings, generally, gamma tACS enhanced ASSR, whereas tACS at lower frequencies resulted in ASSR inhibition. For SSVEPs, modulation was shown to be phase- and frequency-dependent, with congruent tACS and flicker frequencies producing the most reliable effects. Despite methodological heterogeneity and inconsistent results, the reviewed evidence highlights the potential of SSRs as sensitive markers of tACS outcomes. Future studies should aim for well-planned protocols tailored to specific aims and target populations.

## 1 Introduction

Transcranial alternating current stimulation (tACS) is considered a promising non-invasive brain stimulation technique that rhythmically interacts with ongoing brain oscillations in a frequency-specific manner (Antal et al., 2008). By applying a weak sinusoidal current with alternating polarity through the scalp, tACS can entrain endogenous neural activity, potentially modulating sensory, cognitive, and affective processes (Antal and Paulus, 2013; Herrmann et al., 2013; Wischnewski et al., 2023). However, tACS is regarded as a relatively mild intervention, often yielding inconsistent results (Biačková et al., 2024; Chuderski and Chinta, 2024), facing reproducibility issues (Veniero et al., 2017), and being highly susceptible to individual variability (Krause and Cohen Kadosh, 2014; Zanto et al., 2021; Steinmann et al., 2022). Therefore, the technique would greatly benefit from using objective measures to assess brain activity dynamics during and after stimulation.

For this purpose, electroencephalography (EEG) or magnetoencephalography (MEG) is often utilized in tACS studies (Koninck et al., 2023). One particularly promising EEG/MEG paradigm to probe the effects of tACS is the steady-state response (SSR) - a periodic brain response elicited by rhythmic sensory stimulation (Tobimatsu et al., 1999; Brenner et al., 2009; Vialatte et al., 2010). SSRs can be evoked through various modalities, including visual (steady-state visually evoked potentials, SSVEPs), auditory (auditory steady-state responses, ASSRs), and somatosensory (steady-state somatosensory evoked potentials, SSSEPs) stimulation. These responses are highly reliable (Pang and Mueller, 2014; Roach et al., 2019; Fong et al., 2020) and frequency-specific (Tobimatsu et al., 1999; Ding et al., 2006; Roach et al., 2019), making them well-suited for evaluating the frequency-dependent effects of tACS. Studies have shown that tACS may modulate the magnitude and phase consistency of SSRs (Ruhnau et al., 2016a; Baltus et al., 2018; Ahn et al., 2021), potentially reflecting underlying mechanisms such as neural entrainment, resonance, and plasticity (Huang et al., 2021; Agboada et al., 2025).

The translation of tACS into clinical applications depends on identifying reliable biomarkers that index stimulation effects. Biomarkers serve as objective indicators of neuromodulatory efficacy, helping to validate mechanisms, monitor responses, and optimize individualized interventions (Wischnewski et al., 2023). SSRs are particularly attractive in this regard: they are highly reliable, frequency-specific, and can be easily recorded using non-invasive EEG/MEG during periodic sensory stimulation. Moreover, SSRs have been widely used in clinical research as markers of sensory and cognitive dysfunction in conditions such as schizophrenia (Thuné et al., 2016; Schielke and Krekelberg, 2022) or autism spectrum disorder (Pei et al., 2014; Seymour et al., 2020). At the same time, the mechanistic basis of both tACS and SSRs remains debated, and SSRs can theoretically have multiple biomarker roles - they could serve as index of (un)successful entrainment by tACS at individual level, but also be used as a biomarker for behavioral and clinical effects of tACS in the therapeutic context. Thus, a systematic review of how SSRs are modulated by tACS is needed to clarify their potential as biomarkers of stimulation effects.

Although the number of studies investigating SSR neuromodulation by tACS is still relatively limited, the field has grown considerably in recent years. Therefore, the present scoping review aims to synthesize the available tACS–SSR literature, critically evaluate the current empirical findings, identify methodological strengths and limitations, outline key directions and provide guidance for future research.

## 2 Methods

This study was conducted in line with the Preferred Reporting Items for Systematic reviews and Meta-Analyses extension for Scoping Reviews (PRISMA-ScR) (Tricco et al., 2018). Original research articles written in English that addressed tACS effects on SSR were included in the review. Conference papers were considered for inclusion only if they contained sufficient information regarding methods. Only works with a sample size of at least 10 participants were selected to ensure that included studies provide sufficient statistical reliability, since small pilot studies (n < 10) are particularly prone to inflated effect sizes and poor reproducibility (Button et al., 2013).

The eligibility was assessed according to PICO criteria (Table 1): (P) subjects were humans, healthy or diagnosed with neuropsychiatric disorders; (I) steady-state stimulation (visual, auditory and/or somatosensory) and tACS in any frequency were applied; (C) sham-tACS condition or SSR without tACS condition or SSR measurement pre- and post-tACS were used as control procedures; (O) changes in EEG/MEG measures (amplitude, power and/or inter-trial phase-locking) in the frequency range corresponding to steady-state stimulation frequency were assessed.

**Table 1:** PICO criteria for article inclusion.

Population	Humans
Intervention	Steady-state stimulation and tACS
Comparison/control	Sham-tACS condition or SSR without tACS condition or SSR pre- and post-tACS
Outcome	Changes in EEG/MEG measures at the steady-state stimulation frequency

The search was carried out in February 2025. To identify relevant articles, PubMed, Scopus and Web of Science were searched for the keywords *“steady state response,” “steady state evoked potential,” “sensory entrainment,” “auditory steady state response,” “ASSR,” “steady state visual evoked potential,” “steady state visually evoked potential,” “visually evoked steady state response,” “SSVEP,” “steady state somatosensory evoked potential,” “SSSEP”* in combination with *“tACS,” “transcranial alternating current stimulation.”* To ensure that other relevant articles were not missed, bibliographies of the works identified via databases were screened, and an additional non-systematic search was carried out in Google Scholar using keywords *“steady state”* and *“transcranial alternating current stimulation”.*

After removing the duplicates, titles and abstracts of the identified articles were screened to exclude irrelevant works. Methods part and, if necessary, the whole text and supplementary materials of the remaining records were checked to be evaluated according to eligibility criteria (Table 1).

From each included study, the following information was extracted: (1) sample (type, size, age, gender composition); (2) tACS settings (montage, intensity, frequency, duration); (3) control group/condition; (4) experimental design; (5) auditory stimulation settings (frequency, intensity, duration); (6) methods to measure SSR; (7) SSR results; (8) behavioral outcomes. If the publication included multiple studies/experiments, each was analyzed separately.

## 3 Results

**Figure 1:**
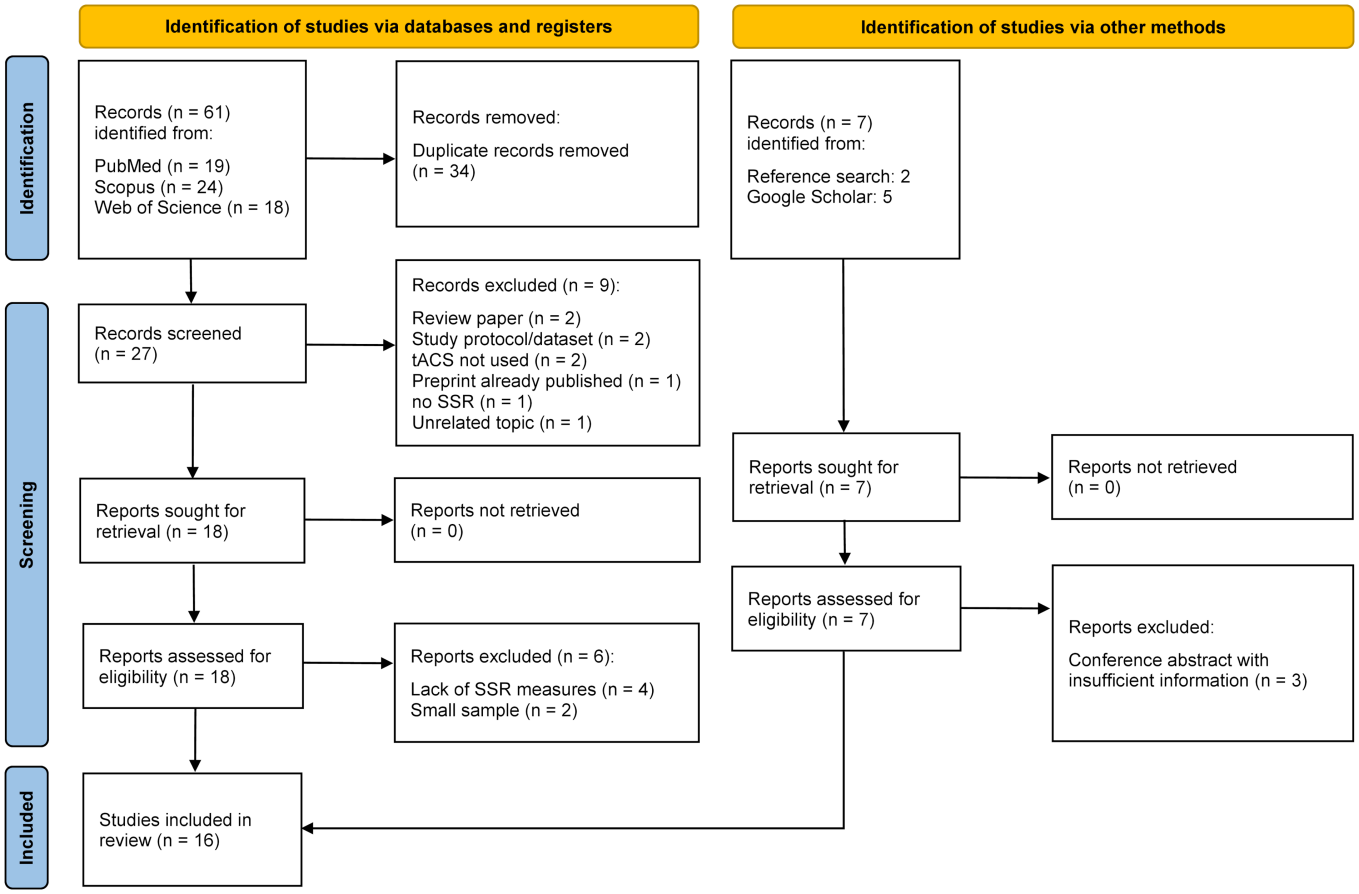
PRISMA flowchart of article search and selection strategy.

In total, the database search yielded 61 entries (Figure 1). Duplicates were removed, and titles and abstracts of 27 articles were screened. Nine papers were excluded after the initial screening: two reviews, two works in which tACS was not utilized, a study protocol, an already-published preprint (i.e., duplicate publication), a paper presenting a dataset, one work where the SSR paradigm was not used, and one study unrelated to the topic. After thoroughly screening the remaining eighteen articles, six works were excluded due to small sample sizes (*n* = 2) or the absence of SSR amplitude, power, or phase-locking analysis (*n* = 4). Seven other papers were found via bibliography search and Google Scholar, of which three were conference abstracts and were excluded due to insufficient information. A total of sixteen articles were included in the present review. Eleven of the included studies evaluated ASSR, and the remaining five assessed SSVEP. The information extracted from the studies is summarized separately for ASSR and SSVEP.

### 3.1 TACS-ASSR studies

#### 3.1.1 Sample characteristics

The information from the reviewed ASSR studies is presented in Table 2. Seven studies assessed tACS-induced ASSR changes only in healthy subjects (Baltus et al., 2018; Hyvärinen et al., 2018; Jones et al., 2020; Wang et al., 2021, 2023; de la Salle et al., 2024; Mockevicius et al., 2025), two studies recruited both healthy subjects and patients either with dyslexia (Marchesotti et al., 2020) or Mal de Débarquement Syndrome (MDdS) (Ahn et al., 2021), the remaining two studies involved only patients with dyslexia (Rufener et al., 2023) or schizophrenia (Ahn et al., 2019). The mean sample size in these studies was 25.1 (SD: 9.6; range: 11–45) with an average age of 26.9 (SD: 10.3; range: 11.59–51.4). Ten studies recruited adult participants, while (Rufener et al., 2023) involved minors.

**Table 2:** Studies that assessed tACS effects on ASSR.

No.	Article	Sample	tACS settings	Control group/condition	Design	Auditory stimulation	Method to measure ASSR	ASSR results	Behavioral outcome
1	Ahn et al. (2019)	Schizophrenia patients *N* = 22 (7f, 15 m) 38.48 ± 10.2 years	Offline continuous tACS; Montage: 5x5cm, between F3 and Fp1, between T3 and P3; 5x7cm, Cz Intensity: 1 mA Frequency: 10 Hz Duration: 20 min	(1) Sham condition (2) Active control tDCS condition	Between-subject 10 sessions of either active, active control or sham (2 per day over 5 days)	Click-trains presented binaurally Frequency: 10 Hz, 20 Hz, 30 Hz, 40 Hz and 80 Hz Intensity: 90 dB SPL Duration: 200 repetitions per frequency, each lasting for 500 ms (15 min in total)	EEG Montage: 128 electrodes Analyzed electrodes: central region Measure: ITPC	40-Hz ASSR increased after 10-Hz tACS, but not after tDCS or sham; no effects of 10-Hz tACS on 10, 20, 30, 80-Hz ASSR	Negative correlation between the change of 40-Hz ASSR after tACS and hallucination scores on day 5; no correlation during 1-week and 1-month follow-ups
2	Ahn et al. (2021)	Mal de Débarquement Syndrome patients *N* = 24 (23 f, 1 m) 53 ± 11.8 years Healthy controls N = 6 (6 f, 0 m), 45.3 ± 6.7 years	Anti-phase or in-phase offline continuous tACS Montage: 10x10cm, midline frontal region and parieto-occipital region, left upper arm Intensity: 1 mA (anti-phase), 2 mA (in-phase) zero-to-peak Frequency: 10 Hz, IAF, IAF + 0.5 Hz, 40 Hz; Duration: 20 min	Healthy controls	Mixed design 10–12 sessions of either anti- or in-phase active (over 3 days)	Click-trains presented binaurally Frequency: 10 Hz, 20 Hz, 30 Hz and 40 Hz Intensity: 90 dB SPL Duration: 100 repetitions per frequency, each lasting 500 ms	EEG Montage: 128 electrodes Analyzed electrodes: 23 central Measure: ITPC	40-Hz ASSR decreased after anti-phase alpha tACS	The degree of ASSR reduction correlated positively with the reduction of symptoms after anti-phase alpha tACS
3	Baltus et al. (2018)	Healthy participants *N* = 26 (14f, 12 m) 24 ± 3.2 years	Offline continuous tACS Montage: 2.5 cm diameter (round), FC5 and TP7/P7 (channel 1), FC6 and TP8/P8 (channel 2) Intensity: 1 mA Frequency: IGF + 4 Hz, IGF-4 Hz (median IGF = 49 Hz) Duration: 2 min pre-task, 5 min during task (7 min total)	No control. Two groups: A and B received tACS at IGF + 4 and IGF-4, respectively	Between-subject Single session of either active A or active B	1,000 Hz AM presented binaurally Frequency: 21–70 Hz Duration: 10s, three repetitions for each stimulus Intensity: not reported	EEG Montage: 32 electrodes Analyzed electrodes: Fz, Cz, Pz Measure: amplitude	ASSR amplitude at stimulation frequency (IGF + 4 Hz or IGF-4 Hz) was greater than ASSR amplitude at IGF after tACS	Significantly shorter auditory gap detection thresholds after IGF + 4 Hz tACS vs. IGF-4 Hz tACS
4	De la Salle et al. (2024)	Healthy participants *N* = 23 (23 m) 21.3 ± 1.9 years	Offline continuous tACS Montage: 5x7cm, T7 and T8 Intensity: individual, 0.1 mA below individual threshold for skin sensation Frequency: 6 Hz and 40 Hz Duration: 20 min	Sham condition	Within-subject Three sessions (one per condition: theta, gamma and sham) separated by a minimum of three days	Click-trains presented binaurally Frequency: 40 Hz Intensity: 80 dB SPL Duration: 150 repetitions, each lasting 500 ms (3 min in total)	EEG Montage: 13 electrodes (Fz, F3, F4, Cz, C3, C4, Pz, P3, P4, TP9, TP10) Analyzed electrodes: Cz Measures: power and ITPC	40 Hz ASSR decreased after 6 Hz tACS; no ASSR change in 40 Hz tACS and sham conditions	No behavioral assessment
5	Hyvärinen et al. (2018)	Healthy participants *N* = 18 (6 f, 12 m); 26.6 ± 4.1 years	Online and offline continuous tACS Montage: 5x7cm, T3 and T4 Intensity: 1.5 mA peak-to-peak Frequency: 6.5 Hz and 12 Hz Duration: 2 blocks for 5 min (12 Hz tACS), 2 blocks for 1 min (6.5 Hz tACS)	(1) Sham stimulation (2) two blocks of 6.5 Hz tACS for 1 min	Within-subject Single session (in which 6.5 Hz, 12 Hz and sham were administered)	Click-trains presented binaurally Frequency: 41 Hz Intensity: 30 dB above hearing threshold Duration: continuous, 2 blocks for 5 min and 1 min each	MEG Montage: 102 magnetometers, 204 planar gradiometers Analyzed channels: right auditory cortex Measure: source power	41 Hz ASSR decreased after 12-Hz tACS; no effect of 6.5-Hz tACS; no differences in ASSR between 12-Hz and 6.5 Hz tACS	No behavioral assessment
6	Jones et al. (2020)	Healthy participants *N* = 45 (26 f, 19 m) 20.9 ± 2.3 years	Offline continuous tACS Montage: 5x5cm, T7 and contralateral cheek Intensity: 1 mA Frequency: 40 Hz Duration: 10 min	(1) Sham condition (2) Active control tDCS condition	Between-subject Single session of either active, active control or sham	Click-trains presented binaurally Frequency: 40 Hz Intensity: 75 dB SPL Duration: 200 repetitions each lasting for 500 ms (7 min in total)	EEG Montage: 14 electrodes (F3, C3, P3, AFz, Fz, FCz, Cz, CPz, Pz, POz, Oz, F4, C4, P4) Analyzed electrodes: all Measures: power and ITPC	40 Hz ASSR power and ITPC increased after 40 Hz tACS; no effect of tDCS	No behavioral assessment
7	Marchesotti et al. (2020)	Dyslexia patients *N* = 15 (13 f, 2 m) 27.4 ± 9 years Healthy controls N = 15 (11 f, 4 m) 25.6 ± 7.8 years	Offline continuous tACS Montage: 4×1 configuration, *π* cm^2^, FTT9h, FCC5h, CPP5h, TPP9h, TTP7h Intensity: individual, 0.6–2 mA peak-to-peak (1.1–1.2 mA peak-to-peak on average) Frequency: 30 Hz and 60 Hz Duration: 20 min	Healthy controls Sham condition	Mixed design Three sessions (one per condition: 60 Hz. 30 Hz or sham) over three days	1,000 Hz AM presented binaurally Frequency: 28 Hz, 30 Hz, 32 Hz, 40 Hz, 58 Hz, 60 Hz and 62 Hz Intensity: 70–75 dB SPL Duration: 40 repetitions per frequency, each lasting 1,500 ms (25 min in total)	EEG Montage: 64 electrodes Analyzed electrodes: FCz (surface) and bilateral auditory cortices (source) Measures: power	30-Hz ASSR increased after 30-Hz tACS in patients; no effect of 60-Hz tACS on 30-Hz ASSR	Significant improvement in phonemic awareness and reading accuracy after 30-Hz tACS in patients
8	Mockevicius et al. (2025)	Healthy participants *N* = 29 (17 f, 12 m) 25.13 ± 3.93 years	Offline continuous tACS Montage: 25 cm^2^ (round), P3 and contralateral cheek Intensity: 2 mA peak-to-peak Frequency: ITF Duration: 20 min	(1) Sham condition (2) tDCS, otDCS conditions	Within-subject One session per condition	Click-trains presented binaurally Frequency: 40 Hz Intensity: 60 dB SPL Duration: 100 repetitions each lasting for 500 ms (~2 min in total)	EEG Montage: 19 electrodes Analyzed electrodes: 6 frontocentral (F3, Fz, F4, C3, Cz, C4) Measures: amplitude and ITPC	No difference in 40-Hz ASSR between conditions	No relationship between ASSR and associative memory changes following tACS
9	Rufener et al. (2023)	Developmental dyslexia patients *N* = 30 (7 f, 23 m), 1 excluded 11.59 ± 2.4 years	Offline continuous tACS Montage: 5x7cm, T7 and T8 Intensity: 1 mA Frequency: 40 Hz Duration: 20 min	Sham condition	Between-subject 10 sessions of either active or sham (two per week over 5 weeks)	1.000 Hz AM presented binaurally Frequency: 30–70 Hz in 1 Hz step Intensity: not reported Duration: 10 s, 3 repetitions for each frequency	EEG Montage: 21 electrodes Analyzed electrodes: Cz Measure: power	ASSR at IGF increased after 40-Hz tACS; no effects during the 4-month follow-up	Improved phonemic skills, no effects during the follow-up; Improved spelling skills observed only during the follow-up
10	Wang et al. (2021)	Healthy participants *N* = 12 (5 f, 7 m) 23.83 ± 0.88 years	Offline continuous tACS Montage: 5x7cm, T8 and T7 Intensity: 2 mA Frequency: 11 Hz Duration: 20 min	Sham condition	Within-subject Single session (in which both active and sham were administered)	Click-trains presented binaurally Frequency: 40 Hz Intensity: 45 dB SPL Duration: 35 repetitions, each lasting 10 s (7 min in total)	EEG Montage: 64 electrodes Analyzed electrodes: F1, Fz, F2, FC1, FCz, FC2, C1, Cz, C2, CP1, CPz, CP2 Measure: power	Significantly stronger decrease in 40-Hz ASSR after sham-tACS vs. 11-Hz tACS	No behavioral assessment
11	Wang et al. (2023)	Healthy participants *N* = 11 (5 f, 6 m) 24.64 ± 0.77 years	Offline continuous tACS; Montage: 5x7cm, T7 and T8 Intensity: 2 mA Frequency: 10 Hz and 40 Hz; Duration: 20 min	Sham condition	Within-subject Four sessions (one per condition: 10 Hz-active, 40 Hz-active, 10 Hz-sham and 40 Hz-sham) separated by at least 7 days	Click-trains presented binaurally Frequency: 40 Hz Intensity: 70 dB SPL Duration: 6 min in total	EEG Montage: 64 electrodes Analyzed electrodes: F1, Fz, F2, FC1, FCz, FC2, C1, Cz, C2, CP1, CPz, CP2 Measure: power	40-Hz ASSR decreased immediately after 10-Hz tACS in real and sham conditions; 40-Hz ASSR remained decreased 30 min after 10-Hz real tACS; No change in 40-Hz ASSR after 40-Hz tACS	No behavioral assessment

Information regarding sample characteristics, tACS settings, control group/condition, design, ASSR acquisition and analysis as well as ASSR and behavioral results is provided.

#### 3.1.2 TACS parameters

Regarding the frequency of stimulation, six studies used gamma (40, 41 Hz or at individual frequencies) tACS (Baltus et al., 2018; Jones et al., 2020; Marchesotti et al., 2020; Ahn et al., 2021; Rufener et al., 2023; Wang et al., 2023; de la Salle et al., 2024), five used alpha (10–12 Hz or at individual frequencies) tACS (Hyvärinen et al., 2018; Ahn et al., 2019, 2021; Wang et al., 2021, 2023) and three applied theta (6, 6.5 Hz or at individual frequencies) tACS (Hyvärinen et al., 2018; de la Salle et al., 2024; Mockevicius et al., 2025). The reviewed studies also applied diverse electrode montages. Eight studies targeted temporal areas, placing electrodes bilaterally (Baltus et al., 2018; Hyvärinen et al., 2018; Wang et al., 2021, 2023; Rufener et al., 2023; de la Salle et al., 2024) or over the left hemisphere (Jones et al., 2020; Marchesotti et al., 2020). Others applied tACS over the left posterior parietal cortex with the return electrode on contralateral cheek (Mockevicius et al., 2025), left frontal and temporo-parietal (Ahn et al., 2019) or frontal and parieto-occipital (Ahn et al., 2021) regions. Looking at the timing of stimulation, ten studies applied continuous tACS separately from ASSR recording (offline), while Hyvärinen et al. (2018) administered tACS and auditory stimulation simultaneously (online). On average, tACS was delivered for 15.5 min (SD: 7.3; range: 1–20) with a fixed intensity ranging from 1 to 2 mA or individually determined intensity – 0.1 mA below the individual skin sensation threshold (de la Salle et al., 2024) or between 0.6 and 2 mA peak-to-peak (Marchesotti et al., 2020).

#### 3.1.3 ASSR parameters

The signal for ASSR assessment was acquired using EEG in 10 studies, whereas Hyvärinen et al. (2018) utilized MEG. Auditory click trains (Hyvärinen et al., 2018; Ahn et al., 2019, 2021; Jones et al., 2020; Wang et al., 2021, 2023; de la Salle et al., 2024; Mockevicius et al., 2025) or 1,000-Hz amplitude modulated tones (Baltus et al., 2018; Marchesotti et al., 2020; Rufener et al., 2023) were used, and sounds were presented binaurally. All studies delivered gamma-band auditory stimulation (30–70 Hz), however, some studies also included lower frequency (10–28 Hz) bands (Baltus et al., 2018; Ahn et al., 2019, 2021). Six studies used short-duration stimuli of 500 ms (Ahn et al., 2019, 2021; Jones et al., 2020; de la Salle et al., 2024; Mockevicius et al., 2025) or 1,500 ms (Marchesotti et al., 2020) with the number of repetitions ranging from 40 to 200 per block; others utilized sounds of 10 s for 3 repetitions (Baltus et al., 2018; Rufener et al., 2023) or 35 repetitions (Wang et al., 2021); conversely, Hyvärinen et al. (2018) used stimuli which continued throughout the whole tACS block, lasting either 1 min or 5 min.

#### 3.1.4 ASSR and behavioral results

The reported findings of ASSR studies are categorized based on the applied frequency of tACS. Among studies that utilized gamma-band tACS, an increase in ASSR at 30 Hz (Marchesotti et al., 2020), 40 Hz (Jones et al., 2020), and at a frequency slightly below or above individual gamma frequency (IGF) (Baltus et al., 2018) was found after applying tACS at an equivalent gamma frequency. In addition, Rufener et al. (2023) reported a stronger ASSR at IGF after 40-Hz tACS. Conversely, no changes in 40-Hz ASSR were reported after 40-Hz tACS in two studies (Wang et al., 2023; de la Salle et al., 2024). Three studies showed that ASSR increase at 30 Hz (Marchesotti et al., 2020), IGF (Rufener et al., 2023) and IGF + 4 Hz (Baltus et al., 2018) was accompanied by enhanced performance in the auditory gap detection task in healthy participants (Baltus et al., 2018) or improvements in language tasks in patients with dyslexia (Marchesotti et al., 2020; Rufener et al., 2023).

When alpha-tACS was applied, suppression of 40-Hz (Ahn et al., 2021; Wang et al., 2023) and 41-Hz (Hyvärinen et al., 2018) ASSR was reported after tACS at 10 Hz (Wang et al., 2023), 12 Hz (Hyvärinen et al., 2018) or when subjects who underwent tACS at 10 Hz, individual alpha frequency (IAF) and IAF + 0.5 Hz were pooled together (Ahn et al., 2021). However, a stronger inhibitory effect of sham tACS on 40-Hz ASSR when compared to 11-Hz tACS was observed in one study (Wang et al., 2021). In addition, Wang et al. (2023) observed a comparable inhibitory effect of both sham and 10-Hz tACS on 40-Hz ASSR, however, only in real tACS condition the effect was present after 30 min following tACS. Conversely, Ahn et al. (2019) showed an opposite effect, with enhanced 40-Hz ASSR after 10-Hz tACS. Two studies that applied alpha tACS also reported the relationship between ASSR changes and behavioral measures: negative correlation was found between ASSR change and auditory hallucination score in schizophrenia (Ahn et al., 2019) and a positive relationship between ASSR reduction and symptom reduction in MdDS (Ahn et al., 2021).

Finally, different results were obtained by three works that used theta tACS. de la Salle et al. (2024) showed that 6-Hz tACS inhibited 40-Hz ASSR, whereas tACS applied at 6.5 Hz (Hyvärinen et al., 2018) and individual theta frequency (ITF) (Mockevicius et al., 2025) showed no effects on 41-Hz and 40-Hz ASSR, respectively. No association between ASSR change following tACS and performance change in associative memory was observed in Mockevicius et al. (2025), whereas other studies did not carry out behavioral assessment.

### 3.2 TACS-SSVEP studies

#### 3.2.1 Sample characteristics

Table 3 contains the information extracted from SSVEP studies. TACS effects on SSVEP were investigated only in healthy participants. On average, 19.6 (SD: 6.2; range: 12–27) subjects participated, with the mean age of 24.7 (SD: 2.4; range: 20.5–26.5) years.

**Table 3:** Studies that assessed tACS effects on SSVEP.

No.	Article	Sample	tACS settings	Control group/condition	Design	Visual stimulation	Method to measure SSVEP	SSVEP results	Behavioral outcome
1	Dowsett et al. (2020)	Healthy participants *N* = 20; 26.5 (22–33) years	Online continuous tACS; Montage: 4 cm diameter, O2 and Cz Experiment 1, group 1: Intensity: 2 mA peak-to-peak; Frequency: 8.3 Hz, 10 Hz, 12.5 Hz Experiment 1, group 2: Intensity: 0.2 mA, 1 mA, 2 mA peak-to-peak; Frequency: 10 Hz Experiment 2: Intensity: 2 mA peak-to-peak; frequency: 10 Hz Duration: 1 min per block, 24 blocks (24 min in total)	Sham condition	Within-subject Experiment 1 Group 1: single session (all frequency conditions in randomized order) Group 2 (separate): single session (all intensity conditions in randomized order) Experiment 2 Single session (two tACS conditions with different flicker frequencies)	Flickering dots Frequency: 10 Hz (experiments 1 and 2), 8.6 Hz (experiment 2) Duration: 6 repetitions, each lasting 5 s, 24 blocks	EEG Montage: 3 electrodes (P3, POz, P4) Analyzed electrodes: all Measure: amplitude	Experiment 1, group 1: Only 10-Hz tACS, but not 8.3-Hz or 12.5-Hz tACS increased 10 Hz SSVEP Experiment 1, group 2: 10-Hz SSVEP increased during 1 mA 10-Hz tACS, but not 0.1 10-Hz tACS; inconclusive results for 0.5 mA 10-Hz tACS Experiment 2: 10-Hz SSVEP, but not 8.6-Hz SSVEP, increased during 10-Hz tACS	No effects of tACS on illusory self-motion
2	Fiene et al. (2020)	Healthy participants *N* = 24 (16 f, 8 m); 25.1 ± 3.3 years	Online intermittent tACS with alternating phase; Montage: 4×1 configuration, 12 mm diameter, over the occipital area; Intensity: 2 mA peak-to-peak; Frequency: 10 Hz; Duration: 6–8 s per repetition, 150 repetitions, 2 blocks (40 min in total)	Sham condition	Within-subject Two sessions (one per condition: active and sham) separated by a minimum of one day	Flickering LED light Frequency: 10 Hz Duration: with tACS, 150 repetitions, each lasting 5.5 s, 2 blocks; without tACS, 100 repetitions, each lasting 5.5 s	EEG Montage: 64 electrodes Analyzed electrodes: O1, O2, POz, Pz Measures: amplitude	10-Hz SSVEP either increased or decreased after 10-Hz tACS depending on the phase shift between tACS and flicker	No behavioral assessment
3	Haslacher et al. (2023)	Healthy participants *N* = 27 (13 f, 14 m), 4 excluded; 26 ± 4 years	Online continuous amplitude-modulated tACS with alternating phase Montage: 5x7cm, AFz and Pz Intensity: 1 mA peak-to-peak Frequency: 6 Hz (40 Hz carrier) Duration: 10 min per block, 30 min in total	Visual flicker without tACS No sham control	Within-subject Single session (in which both tACS and no-tACS were applied)	Flickering grating, green 45° counterclockwise rotation to the left eye, red 45° clockwise rotation to the right eye presented alternatingly Frequency: 6 Hz Duration: continuous, 10 min per block, 3 blocks with tACS, 1 block without tACS	EEG Montage: 64 electrodes Analyzed electrodes: left temporoparietal and right frontal areas Measure: amplitude	6-Hz SSVEP increased if 6-Hz tACS was applied at SSVEP enhancement phase, but reduced if 6-Hz tACS was applied at SSVEP suppression phase	Phase-dependent modulation of binocular perceptual dominance which correlated with SSVEP amplitude modulation
4	Liu et al. (2021)	Healthy participants *N* = 12 (6 f, 6 m); 20.5 ± 2.2 years	Offline continuous tACS Montage: Oz and Cz Intensity: 0.65 mA Frequency: 10 Hz Duration: 20 min	Sham condition	Within-subject Two sessions (one per condition: active and sham) across a minimum of two days	Flickering square Frequency: 10 Hz Duration: 80 repetitions, each lasting 2 s, 1 block pre-tACS, 5 blocks post-tACS	EEG Montage: 64 electrodes Analyzed electrodes: Oz Measure: power	Increased 10-Hz SSVEP immediately after 10-Hz tACS (1st block), but no change in post-tACS blocks 2–5	No tACS effects on Go/No-Go performance and reaction times
5	Ruhnau et al. (2016a)	Healthy participants *N* = 15 (4 f, 11 m), 25.5 years	Online intermittent tACS Montage: 5x7cm, Cz and Oz Intensity: individual, 0.4–1.5 mA (0.613 ± 0.128 mA on average) Frequency: 7 Hz and 11 Hz Duration: 2 s per repetition, 80 repetitions, 4 blocks, 10 min 40 s in total	Sham condition	Within-subject Single session (6 blocks; 1 block per condition)	Flickering ellipse Frequency: 7 Hz or 11 Hz Duration: 80 repetitions, each lasting 2 s, 2 blocks sham, 4 blocks active	MEG Montage: 102 magnetometers and 204 planar gradiometers Analyzed channels: occipital Measures: amplitude, ITPC	After same frequency tACS, 7-Hz and 11-Hz SSVEP ITPC decreased at fundamental (7 Hz / 11 Hz) and second harmonics (14 Hz / 22 Hz); increased amplitude at third (21 Hz / 33 Hz) and fourth harmonics (28 Hz / 44 Hz); increased ITPC at third harmonic for both 7-Hz and 11-Hz SSVEP; increased ITPC at fourth harmonic for 11-Hz SSVEP only	No differences in any conditions in target detection and reaction times

Information regarding sample characteristics, tACS settings, control group/condition, design, SSVEP acquisition and analysis as well as SSVEP and behavioral results is provided.

#### 3.2.2 TACS parameters

Looking at the frequencies, four studies applied alpha-band (8.3–12.5 Hz) tACS (Ruhnau et al., 2016a; Dowsett et al., 2020; Fiene et al., 2020; Liu et al., 2021), theta (6–7 Hz) tACS was used in two works (Ruhnau et al., 2016a; Haslacher et al., 2023). Haslacher et al. (2023) used amplitude-modulated 6-Hz tACS with a 40-Hz carrier. In addition, two studies introduced an alternation of tACS phase relative to the visual flicker (Fiene et al., 2020; Haslacher et al., 2023). Two studies applied tACS online intermittently (Ruhnau et al., 2016a; Fiene et al., 2020) with tACS and visual flicker combined into simultaneous repetitions. Fiene et al. (2020) presented 150 repetitions per block, each lasting 6–8 s. Ruhnau et al. (2016a) delivered 2-s tACS pulses in 80 repetitions per block. Two studies used online continuous tACS divided into blocks lasting 1 min (Dowsett et al., 2020) or 10 min (Haslacher et al., 2023). Liu et al. (2021) used an offline continuous tACS with a duration of 20 min. Fixed intensities ranged from 0.2 to 2 mA, while individually determined intensities were set between 0.4 to 1.5 mA (Ruhnau et al., 2016a). As per electrode montages, occipital (Ruhnau et al., 2016a; Dowsett et al., 2020; Fiene et al., 2020; Liu et al., 2021) and parietal (Haslacher et al., 2023) areas were targeted. The occipital montage consisted of two electrodes in central/occipital areas (Dowsett et al., 2020; Liu et al., 2021; Ruhnau et al., 2016a) or 4×1 configuration (Fiene et al., 2020).

#### 3.2.3 SSVEP parameters

Four studies recorded EEG and one used MEG (Ruhnau et al., 2016a). Participants watched flickering shapes or patterns on the screen (Ruhnau et al., 2016a; Dowsett et al., 2020; Liu et al., 2021; Haslacher et al., 2023) or a flickering light (Fiene et al., 2020). Dowsett et al. (2020) introduced flickering to optic flow patterns or random dot motion visual stimuli, whereas Haslacher et al. (2023) incorporated the steady-state aspect into a binocular rivalry task. Four studies used short stimuli of 2 s (Ruhnau et al., 2016a; Liu et al., 2021), 5 s (Dowsett et al., 2020) or 5.5 s (Fiene et al., 2020) presented from 6 to 150 times per block, whereas Haslacher et al. (2023) delivered continuous 10-min stimuli. Alpha (Ruhnau et al., 2016a; Dowsett et al., 2020; Fiene et al., 2020; Liu et al., 2021) and/or theta (Ruhnau et al., 2016a; Haslacher et al., 2023) stimulation frequencies were used. Three studies used flicker frequencies congruent with tACS frequency (Fiene et al., 2020; Liu et al., 2021; Haslacher et al., 2023), while two studies applied both congruent and incongruent flicker frequencies (Ruhnau et al., 2016a; Dowsett et al., 2020).

#### 3.2.4 SSVEP and behavioral results

An increase in 10-Hz SSVEP during/after 10-Hz tACS was reported in two works (Dowsett et al., 2020; Liu et al., 2021). In addition, Dowsett et al. (2020) showed that no effects were present when tACS and SSVEP were delivered at different frequencies. Fiene et al. (2020) demonstrated that 10-Hz SSVEP can be both enhanced and inhibited by 10-Hz tACS, depending on the phase shift between tACS and the flicker. A similar phase-dependent modulation of 6-Hz SSVEP applying 6-Hz tACS was reported by Haslacher et al. (2023). Ruhnau et al. (2016a) used tACS and SSVEP at 7-Hz and 11-Hz and showed a differential modulation of SSVEP amplitude and phase-locking for different harmonics. Importantly, these effects were observed only when the same frequency was used for the flicker and tACS. Haslacher et al. (2023) showed a relationship between changes in SSVEP and binocular stimulus dominance ratio, while the other four studies did not report any significant behavioral effects.

## 4 Discussion

The present review shows that research combining tACS with SSR paradigms has grown substantially in recent years offering a unique opportunity for probing oscillatory brain dynamics. Since the last review on tES effects on ASSR (Griskova-Bulanova et al., 2020), the number of works investigating the modulation of ASSR using tACS has increased from 3 to 11. In addition, five tACS-SSVEP studies were featured in this review. TACS-SSR is a promising paradigm that could provide valuable insights into tACS neurophysiological and behavioral effects, yet the variability in reported effects underscores the need for a clearer mechanistic framework.

A key point concerns the neurophysiological basis of both tACS and SSRs. While entrainment of endogenous oscillations has often been proposed as the common mechanism underlying both phenomena (Thut et al., 2011), accumulating evidence suggests a more complex picture. For tACS, recent critical review of neurocognitive, physiological, and biophysical effects highlights entrainment of endogenous oscillations as a central mechanism but also presents evidence of additional effects, including shifts in neural spike timing, alterations in interregional coherence and connectivity, and even broader homeostatic or metabolic changes (Wischnewski et al., 2023). Similarly, the after-effects of tACS have been attributed to multiple forms of plasticity, such as spike-timing-dependent plasticity, spike-phase and oscillation coupling, homeostatic and state-dependent modulation (Agboada et al., 2025).

The neurophysiological basis of SSRs is likewise not unitary (Bánki et al., 2022). Several works showed a high correspondence between recorded SSRs and a synthesized waveform of linearly summated transient evoked responses to discrete stimuli (Azzena et al., 1995; Bohórquez and Özdamar, 2008; Capilla et al., 2009, 2011), suggesting that SSRs may rely on the same underlying mechanism as sensory evoked potentials and occur independently of the ongoing intrinsic oscillations. However, a larger body of evidence favors the entrainment of ongoing activity via resonant frequencies (Johnson et al., 2024) or the activation of separate rhythms by stimulation (Welle and Contreras, 2016; Duecker et al., 2021). Moreover, SSRs (1) reflect changes in neural activity in neuropsychiatric conditions (Yamasaki, 2021; Grent-‘t-Jong et al., 2023) or due to pharmacological modulation (Bale et al., 2005; Sullivan et al., 2015), (2) are associated with behavioral measures (Richard et al., 2020; Parciauskaite et al., 2021), and (3) are stronger and more synchronized at frequencies matching intrinsic individual-specific frequencies (Zaehle et al., 2010; Notbohm et al., 2016). Furthermore, prolonged rhythmic sensory stimulation has shown promise in modulating neurobiological (Martorell et al., 2019; Rodrigues-Amorim et al., 2024), neurophysiological (Lin et al., 2021; Pinardi et al., 2024) and behavioral (Sharpe et al., 2020; Cimenser et al., 2021) outcomes in both animals and humans. Together, these findings point to the potential interaction of periodic sensory stimulation with intrinsic brain oscillations.

Motivated by the notion of shared neural entrainment mechanisms hypothesized for both tACS and SSRs (Thut et al., 2011), several of the reviewed studies combined SSRs with tACS, aiming to investigate various factors related to tACS effects mechanistically. Among ASSR studies, low-frequency tACS was generally used to probe cross-frequency interactions (Hyvärinen et al., 2018; Wang et al., 2021, 2023; Mockevicius et al., 2025), while gamma tACS was utilized to study how tACS affects endogenous oscillations at a matched frequency (Jones et al., 2020; Wang et al., 2023). SSVEP paradigm was employed to evaluate the impact of tACS intensity (Dowsett et al., 2020), frequency (Ruhnau et al., 2016a; Dowsett et al., 2020; Liu et al., 2021), and phase (Fiene et al., 2020; Haslacher et al., 2023) on rhythmic brain activity. In addition, among the reviewed studies, SSRs were evaluated as biomarkers, assessing the link between SSRs and behavioral effects of tACS, including symptom severity in patients with MdDS (Ahn et al., 2021), schizophrenia (Ahn et al., 2019), and dyslexia (Marchesotti et al., 2020; Rufener et al., 2023) or sensory task performance in healthy participants (Baltus et al., 2018).

Given that tACS is oscillatory in nature, the frequency of stimulation is a fundamental parameter. The selection of tACS frequency among the reviewed studies mainly differed based on goals and the putative mechanisms of action. In ASSR studies, gamma tACS was delivered to enhance synchronized neural activity at specific gamma-range frequencies. However, the results are mixed as studies showed either a predicted increase (Baltus et al., 2018; Jones et al., 2020; Marchesotti et al., 2020; Rufener et al., 2023) or no change (Wang et al., 2023; de la Salle et al., 2024). Due to the general inhibitory role of alpha activity (Jensen and Mazaheri, 2010; Mathewson et al., 2011), potentially mediated via alpha-gamma cross-frequency interactions, alpha tACS was primarily selected to suppress gamma synchronization. While this alpha tACS effect was reported in MdDS patients (Ahn et al., 2021) as well as healthy participants (Hyvärinen et al., 2018; Wang et al., 2023), an increase in ASSR was also shown in schizophrenia patients (Ahn et al., 2019). de la Salle et al. (2024) expected increased gamma ASSR due to theta tACS based on theta-gamma phase-amplitude coupling, but the authors observed an ASSR reduction. Other studies showed no theta tACS effects on gamma ASSR (Hyvärinen et al., 2018; Mockevicius et al., 2025). Among the studies assessing SSVEP, the general aim was to enhance theta or alpha activity using a congruent tACS frequency; however, the effect was shown to depend on the phase lag between visual flicker and tACS delivered online (Fiene et al., 2020; Haslacher et al., 2023). When the frequencies of visual flicker and tACS did not match, no change in SSVEP was reported (Ruhnau et al., 2016a; Dowsett et al., 2020).

Overall, the reviewed findings suggest that tACS effects on SSRs depend on sensory and electrical stimulation frequencies. However, there is a substantial variability in the reported findings, especially among ASSR studies, which likely arose due to differences in methodology and sample characteristics. When gamma tACS was used, promising results were reported in studies with specific *a priori* hypotheses about disturbances of gamma oscillations in clinical groups or the relationship of gamma activity with targeted sensory processing. In two studies, the applied approaches were based on the hypothesis of reduced peak frequency of gamma oscillations in dyslexia. Marchesotti et al. (2020) showed an increase in 30-Hz ASSR after 30-Hz tACS, while Rufener et al. (2023) reported an increase in both IGF and response at IGF after 40-Hz tACS. Improvements in language task performance were also shown in both studies. In addition, Baltus et al. (2018) hypothesized that IGF can be related to auditory temporal acuity and showed that tACS applied at a frequency slightly above IGF increased ASSR at the corresponding frequency and improved auditory gap detection performance in healthy subjects. Other studies targeted the classical 40-Hz ASSR using the same frequency tACS in healthy young subjects. Of them, only Jones et al. (2020) reported an increase in ASSR, while in two studies ASSR remained unchanged (Wang et al., 2023; de la Salle et al., 2024). The latter two studies are characterized by small sample sizes (23 and 11 subjects), which could have contributed to a lack of significant effects. TACS is a relatively mild non-invasive intervention that is generally characterized by modest to moderate effect sizes (Grover et al., 2023). In addition, healthy participants are expected to be more resilient to external perturbations (Hall et al., 2020), which is likely applicable to ASSR due to its high individual stability (Van Eeckhoutte et al., 2018; Roach et al., 2019). Due to these factors, larger sample sizes may be needed to detect ASSR changes after matched frequency tACS.

Similarly, studies using low-frequency tACS and gamma ASSR also showed inconsistent outcomes. Patient sample characteristics may explain the differences in clinical studies using alpha tACS. Given that MdDS is characterized by gamma hypersynchrony, alpha entrainment by tACS may have normalized excessive gamma activity, potentially due to its inhibitory influence (Ahn et al., 2021). Conversely, since schizophrenia patients exhibit inadequate oscillatory activity in both alpha and gamma bands (Uhlhaas et al., 2008), alleviating alpha disturbances may have also contributed to improved gamma synchronization due to alpha-gamma interactions (Jensen and Mazaheri, 2010) as evidenced by increased ASSR (Ahn et al., 2019).

Theta tACS was used in three studies, showing either reduced (de la Salle et al., 2024) or unchanged (Hyvärinen et al., 2018; Mockevicius et al., 2025) ASSR. Montage choice may have contributed to mixed results. Despite the overall low focality of tACS, the induced electric field strength differs substantially across configurations (Guidetti et al., 2022), and this variability likely contributes to inconsistent outcomes. Hyvärinen et al. (2018) and De la Salle et al. (2024) applied theta tACS over bilateral temporal regions, whereas Mockevicius et al. (2025) targeted posterior parietal cortex. Since ASSR is mainly generated in the auditory cortex (Pantev et al., 1996), ASSR may have been less sensitive to tACS applied over a more distant location from its primary source. Also, authors highlight the role of individual variability as contributing to insignificant findings at the group level (Mockevicius et al., 2025). Meanwhile, the study by Hyvärinen et al. (2018), contains methodological limitations that hinder clear interpretation of the findings. Online theta and alpha tACS were applied for different durations - 1 min and 5 min, respectively. Although the authors partly accounted for this by comparing ASSR during theta and alpha tACS in 1-min time window, ASSR during alpha tACS was compared to no-tACS ASSR over the whole 5-min window, likely resulting in different signal-to-noise ratios and differences in the overall state. Both tACS conditions showed ASSR suppression descriptively, but only alpha tACS produced a statistically significant effect. However, ASSR did not differ between theta and alpha tACS conditions, suggesting no frequency-specific effects on ASSR (Hyvärinen et al., 2018).

Studies combining visual flicker with tACS were more methodologically homogeneous and reported consistent findings. All studies recruited only healthy participants and targeted posterior areas (occipital or parietal) using low frequency (alpha and/or theta) tACS, unanimously showing significant tACS effects when flicker and tACS frequencies were matched. In addition, four out of five studies used online tACS combined with visual stimulation. Three works used artifact-removal methods (Ruhnau et al., 2016a; Dowsett et al., 2020; Haslacher et al., 2023), whereas Fiene et al. (2020) analyzed uncontaminated parts of the signal immediately following online tACS. These approaches allowed for more precise and robust mechanistic investigations, such as phase-dependence of tACS-SSVEP outcomes (Fiene et al., 2020; Haslacher et al., 2023). In addition, given that tES effects are network-activity-dependent (Fertonani and Miniussi, 2017), online tACS in SSVEP studies could have contributed to more pronounced and thus more consistent effects when compared to ASSR studies that applied tACS offline. While analyzing ASSR recorded during simultaneous tACS application could be challenging due to inherently weaker gamma response as compared to low-frequency SSVEP, assessing ASSR immediately following online tACS (Fiene et al., 2020) or using non-overlapping tACS and ASSR frequencies and their harmonics (Hyvärinen et al., 2018) may be promising strategies for future online tACS-ASSR studies.

However, it is essential to mention that only one tES-SSVEP study reported significant changes in behavioral measures (Haslacher et al., 2023), likely due to the generally mechanistic nature of the studies. Firstly, only healthy participants were recruited. It has been suggested that tACS may be more beneficial for patients with psychiatric disorders as compared to healthy individuals (Lee et al., 2022). In line with this, among ASSR studies, significant behavioral effects of tACS were mainly evident in clinical populations (Ahn et al., 2019, 2021; Marchesotti et al., 2020; Rufener et al., 2023), with only Baltus et al. (2018) reporting changes in auditory perception in healthy participants. These findings fit the inverted U-shaped relationship between baseline neural activity and stimulation outcomes (Rufener et al., 2016; Ruhnau et al., 2016b; Tseng et al., 2018; Krause et al., 2022). Namely, tACS appears most effective when intrinsic oscillatory activity is suboptimal, as observed in clinical populations with reduced or dysregulated neural synchrony. In contrast, when baseline activity in healthy brains is already near optimal, tACS may have little or even disruptive effects. This principle may help reconcile why tACS robustly enhanced ASSRs in dyslexia or schizophrenia, yet yielded inconsistent effects in healthy participants. Secondly, in two studies (Fiene et al., 2020; Ruhnau et al., 2016a), the intermittent pattern of tACS might have been inadequate for behavioral modulation as it may require longer-lasting entrainment (Strüber et al., 2015). Furthermore, the differences in behavioral tasks should be taken into consideration. For example, Haslacher et al. (2023) studied binocular dominance and observed its relationship with SSVEP modulation. Conversely, Liu et al. (2021) analyzed Go/NoGo performance, showing no changes after tACS. While the former assessed lower-level perceptual processing, the Go/NoGo task in the latter study relies on multiple higher-order cognitive processes (e.g., inhibitory control, Bokura et al. (2001)), potentially making it less sensitive to tACS applied over the occipital region.

Given the complex and still-explored mechanisms underlying both tACS effects and SSRs, the heterogeneous findings are no surprise. At a neurophysiological level, tACS may interact with SSRs in multiple ways: (1) by entraining intrinsic neural oscillators at the stimulation frequency, thereby enhancing resonance phenomena, supported by cross-frequency interactions in ASSR studies and the reported relationships with behavioral measures; (2) by modulating the amplitude of repetitive evoked responses without necessarily engaging endogenous oscillators, which is in line with more consistent results when tACS frequency and phase were matched with those of SSRs; or (3) by periodically biasing membrane potential fluctuations, which can alter responsiveness to incoming rhythmic sensory input. Distinguishing these mechanisms remains challenging, yet it is critical for interpreting whether reported SSR modulations index genuine entrainment or altered evoked responses.

Taken together, the reviewed evidence suggests that tACS exerts neurophysiological effects which may be reflected in changes to SSR amplitude, power, or phase-locking, likely indexing the entrainment of intrinsic oscillations or the modulation of evoked responses. These tACS-induced changes may manifest behaviorally, e.g., as improved clinical symptoms, auditory gap detection or reading performance, which appear to be more consistent when tACS restores or enhances oscillatory activity in populations with atypical baseline activity (e.g., dyslexia, schizophrenia). These differences highlight that tACS effects are not uniform but depend on the interplay between stimulation parameters and the brain state of the participant. This mechanistic perspective can explain why SSRs may serve as both biomarkers of neural entrainment and predictors of behavioral outcomes, adding to their translational value.

The findings reviewed in the present work contribute to the existing literature by showing that tACS neurophysiological and behavioral effects depend on both extrinsic (methodology-related) and intrinsic (brain-activity-related) factors. Accordingly, future studies should focus on adapting tACS-SSR procedures to the specific aims and participant populations. When investigating tACS mechanisms of action using SSRs, online tACS would be preferred due to the possibility of applying precise experimental manipulations. Offline tACS may be suitable when assessing its potential as a biomarker of tACS-induced behavioral changes. In particular, tACS protocols aiming to improve a specific sensory or cognitive domain may include the combination of tACS with online behavioral tasks (e.g., Bjekić et al., 2022). Therefore, SSRs would be limited to offline use in these approaches. However, based on the reviewed findings, offline tACS may require a careful and theoretically grounded selection of protocol parameters as well as higher sample sizes.

In summary, this review advances the field by summarizing up to date literature on tACS-SSR interaction. This review synthesizes cross-study patterns and identifies three converging insights: (1) congruence between sensory and electrical stimulation frequencies is the most reliable determinant of tACS effects on SSRs; (2) variability in baseline oscillatory dynamics is expected to modulate the effects of tACS-SSR applications, which may result in differential outcomes across healthy and clinical populations; and (3) methodological differences, particularly online vs. offline designs, account for the variability and heterogeneity of the observed effects. Together, these insights advance a conceptual framework for using SSRs not only as mechanistic probes of entrainment but also as candidate biomarkers of tACS efficacy.

## Data Availability

The original contributions presented in the study are included in the article/supplementary material, further inquiries can be directed to the corresponding author.
